# Noggin promotes osteogenesis in human adipose-derived mesenchymal stem cells via FGFR2/Src/Akt and ERK signaling pathway

**DOI:** 10.1038/s41598-024-56858-w

**Published:** 2024-03-20

**Authors:** Karolina Truchan, Anna Maria Osyczka

**Affiliations:** Department of Cell Biology and Imaging, Institute of Zoology and Biomedical Research, Jagiellonian University, Gronostajowa St. 9, 30-387 Kraków, Poland

**Keywords:** Growth factor signalling, Mesenchymal stem cells, Stem-cell differentiation, Stem-cell biotechnology

## Abstract

The balance between Noggin and bone morphogenetic proteins (BMPs) is important during early development and skeletal regenerative therapies. Noggin binds BMPs in the extracellular space, thereby preventing BMP signaling. However, Noggin may affect cell response not necessarily through the modulation of BMP signaling, raising the possibility of direct Noggin signaling through yet unspecified receptors. Here we show that in osteogenic cultures of adipose-derived stem cells (ASCs), Noggin activates fibroblast growth factor receptors (FGFRs), Src/Akt and ERK kinases, and it stabilizes TAZ proteins in the presence of dexamethasone. Overall, this leads ASCs to increased expression of osteogenic markers and robust mineral deposition. Our results also indicate that Noggin can induce osteogenic genes expression in normal human bone marrow stem cells and alkaline phosphatase activity in normal human dental pulp stem cells. Besides, Noggin can specifically activate FGFR2 in osteosarcoma cells. We believe our findings open new research avenues to further explore the involvement of Noggin in cell fate modulation by FGFR2/Src/Akt/ERK signaling and potential applications of Noggin in bone regenerative therapies.

## Introduction

Noggin is classified as one of the extracellular inhibitors of bone morphogenetic proteins (BMPs) and it binds to BMPs in the extracellular space to prevent them from binding to BMP receptors, consequently inhibiting BMP signaling^1^. Noggin is a glycosylated homodimer protein with a cysteine knot and heparin-binding segments^2^, and it can be retained at the cell surface by heparan sulfate proteoglycans (HSPGs). The latter regulate the diffusion of Noggin and thus enable the formation of a BMP activity gradient^3,4^. BMPs are broadly studied for their potential of inducing chondro- and osteogenesis both in vitro and in vivo^5^. Among more than 20 known members of the BMP family, recombinant human BMP-2 and BMP-7 have been initially approved for restricted clinical use in open fractures of long bones, non-unions, and spinal fusion^6^. Notably, despite BMP-2 applications have raised several concerns^7^, BMP-2 remains the most commonly explored growth factor for delivery e.g., for spinal fusion^8^. The extensive BMP-2 off-label applications are though steadily growing, and the reports of clinical side effects associated with such off-label BMP use include e.g., the inflammation of adjacent tissues, wound complications such as hematoma formation, neurological disorders and, in cervical procedures, life-threatening complications of compromised airways^9–15^. Besides, BMP-2 is reportedly less effective in human than in rodent cells^16–19^ and the cellular response to BMP-2 is age-related^20^. Similar concerns relate to the clinical use of BMP-7, which has been withdrawn from the market^21^. Abnormal BMP signaling has also been observed in a subset of joint disorders known as spondyloarthropathies^22–25^. Therefore, potential therapeutic approaches for the latter conditions now include the targeting of BMPs through agents like Noggin and other BMP antagonists, with the aim of attenuating articular cartilage degeneration by preventing BMP-induced joint inflammation and endochondral ossification of chondrocytes^26,27^.

The lack of the Noggin gene during early development results in a lethal phenotype with impaired neural tube closure, deficient somite development and limbs malformations^28^. Mutations in the human Noggin gene are associated with joint fusions in multiple synostoses syndromes and proximal symphalangism^29^. On the contrary, the suppression of Noggin expression improves BMP-mediated osteogenesis in several mouse and human cells^30–32^. Other studies show that Noggin suppression decreases BMP-induced osteogenesis in human mesenchymal stem cells (MSCs)^33^ and Noggin inactivation causes osteopenia in mice^34^. Recent reports demonstrate the positive role of Noggin in osteogenesis, with Noggin treatment resulting in osteogenic differentiation of canine dental pulp stem cell (DPSC) cultures and subsequent mineralization of their extracellular matrix^35^. The addition of Noggin to osteogenic media was also reported to increase ALP activity in human osteoblast cultures^36^, and this study suggested BMP-2 and Noggin receptor binding competition.

Some reports on the actions of Noggin beyond BMP inhibition have recently emerged. Noggin has been shown to induce adipogenesis in both rat and human MSC cells by Pax-1 activation and elevated levels of Noggin protein were detected in serum of obese individuals^37^. Under high-glucose conditions, Noggin could inhibit apoptosis and promote insulin secretion in pancreatic beta cells through the inhibition of SMAD signaling^38^. Other studies suggest that Noggin may act independently of BMPs, presumably in a manner similar to Gremlin, another BMP antagonist^4^, which binds to VEGFR-2^39^. Thus, Noggin appears to function as a molecular switch in determining the fate of adipose stem cells, transmitting signals to these and other cells through yet unidentified cell-surface receptors.

Understanding Noggin intracellular signaling in adipose-derived stem cells (ASCs), whether alone or in relation to BMPs, is clinically relevant, especially that ASCs now serve as an alternative to bone marrow source of stem cells for cellular therapies in rheumatology and orthopedics^40,41^. Since their discovery in 2001^42^, ASCs have been shown to differentiate into osteoblasts and other non-fat cell phenotypes^42–44^. The advantages of ASCs over bone marrow stem cells (BMSCs), rely in higher isolation efficacy and less invasive harvesting procedures compared to bone marrow^45^. The use of Noggin to stimulate differentiation of ASCs into osteoblasts presents an attractive alternative to other growth factors (including BMPs) broadly used in tissue engineering and thus it may lead to several new tissue engineering strategies with ASCs and other human MSC types.

In the present study, we dissect the mechanisms by which Noggin protein induces osteogenesis in human adipose-derived mesenchymal stem cells. We report that Noggin, when added to osteogenic medium containing dexamethasone, prompts osteogenesis in adult human MSCs and advances osteogenic differentiation of human ASC cultures. We also reveal for the first time the Noggin-induced FGFR2/Src/Akt/ERK intracellular signaling pathway, which may have broader biological implications, not limited to ASC-related bone therapies, but extended to several other biological processes involving Noggin actions.

## Results

### Noggin increases ALP activity and expression of early osteogenic markers in adult human mesenchymal stem cells from different tissues

**Figure 1:**
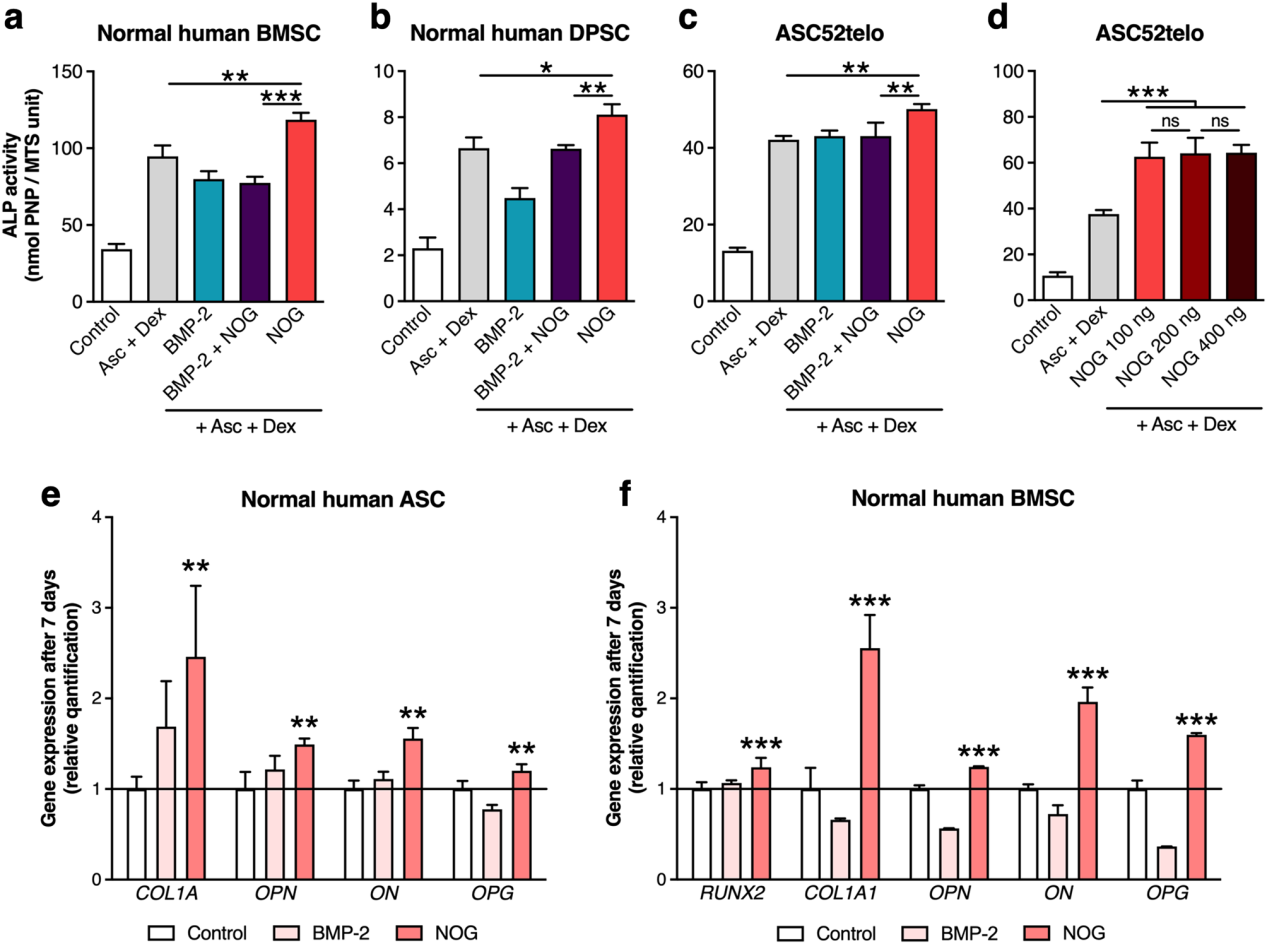
Noggin protein increases alkaline phosphatase (ALP) activity and early osteogenic genes expression in adult human mesenchymal stem cells from different tissues. Alkaline phosphatase (ALP) activity after 7-d culture of (**a**) normal human bone marrow stromal cells (BMSCs), (**b**) normal human dental pulp stem cells (DPSCs) and (**c**) human immortalized adipose-derived stem cell line (ASC52telo). Cells were treated with either 100 ng/ml recombinant human Noggin (NOG) or 100 ng/ml recombinant human bone morphogenetic protein 2 (BMP-2), or both (BMP-2 + NOG), in osteogenic medium containing ascorbic acid (Asc) and dexamethasone (Dex). (**d**) ALP activity after 7-d culture of ASC52telo cells in osteogenic medium with different Noggin doses (100–400 ng/ml). (**a–d**) Control represents cells maintained in standard growth medium. Relative mRNA levels (qPCR) of selected osteoblastic markers in (**e**) normal human ASCs and (**f**) normal human BMSCs continuously treated with Noggin (NOG) or BMP-2 for 7 days in osteogenic medium. Relative quantification to control cells cultured in osteogenic medium. (**a–f**) Average values ± SD are plotted. One-way ANOVA tests, *p < 0.05, **p < 0.001, ***p < 0.0001, ns—not significant, relative to respective control or between marked groups.

Given that some reports indicate that Noggin treatment may result in increased alkaline phosphatase (ALP) activity of human osteoblasts^36^, we investigated the effect of Noggin on ALP activity of adult human mesenchymal stem cells derived from three different tissues, i.e., bone marrow, dental pulp, and adipose tissue. Noggin effects on ALP activity and osteogenic gene expression were compared to BMP-2 effects alone or combined with Noggin. Please note that Noggin effects on osteogenic phenotypes were investigated by adding Noggin and/or BMP-2 to standard osteogenic medium containing ascorbic acid (Asc) and dexamethasone (Dex). The terms “Noggin alone” or “Noggin treatment” refer to Noggin being the only factor added to the aforementioned osteogenic media (Noggin effects with or without dexamethasone are dissected further in Fig. 6). In all three studied stem cell populations Noggin alone significantly increased ALP activity after 7-day culture compared to standard osteogenic media (Fig. 1a–c), but this was not observed upon BMP-2 or combined Noggin and BMP-2 treatment. The significant increase of ALP activity in ASC cells upon Noggin treatment was observed at 100 ng/ml Noggin dose and this was not further enhanced by higher Noggin doses (Fig. 1d). In human primary ASCs and BMSCs (Fig. 1e,f), Noggin also increased gene expression of early osteogenic markers i.e., collagen type I (*COL1A1*), osteopontin (*OPN*), osteonectin (*ON*), osteoprotegerin (*OPG*) and runt-related transcription factor 2 (*RUNX2*). Thus, in addition to human osteoblasts^36^, Noggin treatment can also result in increased ALP activity of adult human mesenchymal stem cells derived from bone marrow, dental pulp and adipose tissue as well as increased osteogenic markers expression in normal human ASCs and BMSCs.

### Noggin treatment results in increased expression of osteogenic markers in ASC cells and their extracellular matrix mineralization

**Figure 2:**
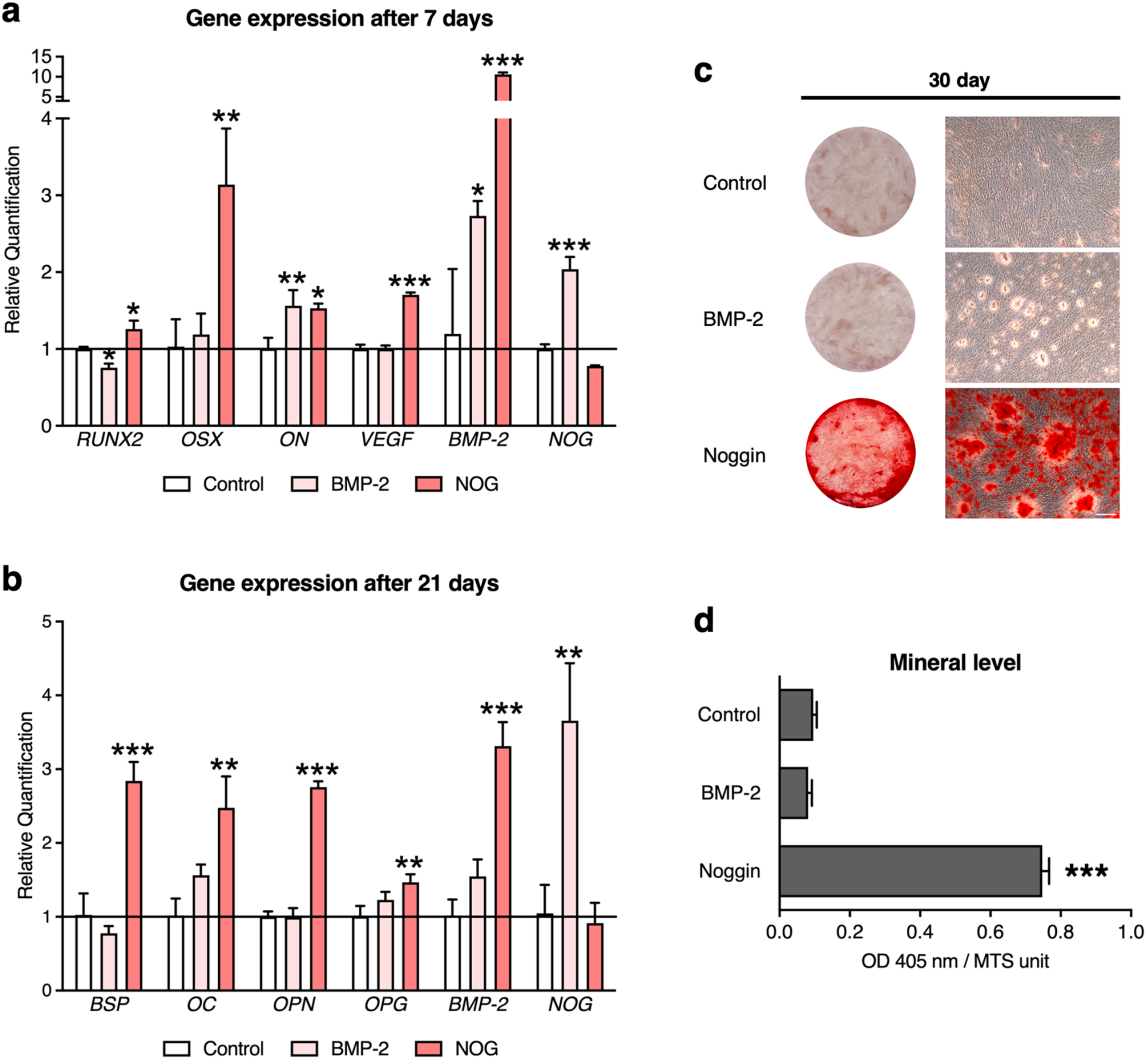
Noggin promotes the osteogenic markers expression and the deposition of mineralized matrix in ASC cultures. Relative mRNA levels (qPCR) of selected osteoblastic markers in ASC cells continuously treated with Noggin (NOG) or BMP-2 for (**a**) 7 days or (**b**) 21 days in osteogenic medium. Relative quantification to control cells cultured in osteogenic medium. (**c**) Extracellular matrix mineral levels (Alizarin Red S staining) in ASC cells after 30-day continuous treatment with either Noggin or BMP-2 in osteogenic medium. Scale bar represents 100 μm. (**d**) Colorimetric quantification of Alizarin Red S dye extracted from stained cells; results normalized to viable cell number (MTS assay). (**a–d**) Control represents cells cultured in osteogenic medium containing ascorbic acid, dexamethasone and β-glycerophosphate. Average values ± SD are plotted. One-way ANOVA tests, *p < 0.05, **p < 0.001, ***p < 0.0001 relative to a control group.

Given that Noggin increased ALP activity, the early marker of osteogenic events, in all studied human MSC populations (Fig. 1a–c) and mRNA levels of early osteogenic markers in both normal human ASCs and BMSCs (Fig. 1e,f), we explored the osteogenic effects of Noggin in adipose-derived MSCs, assuming their broadening clinical applicability. Since Noggin increased osteogenic markers in normal human ASCs, further studies were carried out with immortalized human adipose-derived stem cell line (briefed ASC). The latter, treated for 7 days with Noggin, increased mRNA levels of *RUNX2*, osterix (*OSX*), osteonectin (*ON*) and vascular endothelial growth factor (*VEGF*) (Fig. 2a) compared to control cells maintained in osteogenic medium only. The continuous Noggin treatment for 21 days resulted in increased mRNA levels of bone sialoprotein (*BSP*), osteocalcin (*OC*) and osteopontin (*OPN*), and bone remodeling-associated osteoprotegerin (*OPG*) (Fig. 2b). ASCs treated with BMP-2 up-regulated mRNA expression of Noggin (*NOG*), whereas treatment with Noggin resulted in up-regulation of *BMP-2* expression (Fig. 2a,b), confirming the feedback loop between BMP-2 and Noggin. Notably, we did not detect BMP-2 protein in the culture medium after 7-day culture with Noggin or in control cells, whereas BMP-2 treatment resulted in BMP-2 detection (Supplementary Fig. 1). Notably, the continuous Noggin treatment of ASC cultures for 30 days resulted in significant mineral deposition compared to BMP-2 treated cells or control cells cultured in osteogenic medium (Fig. 2c,d). These results strongly indicate that Noggin is capable to stimulate both early and late ASC osteogenesis that leads to mineralized matrix deposition.

### Noggin treatment results in the activation of ERK1/2 and Akt intracellular kinases

**Figure 3:**
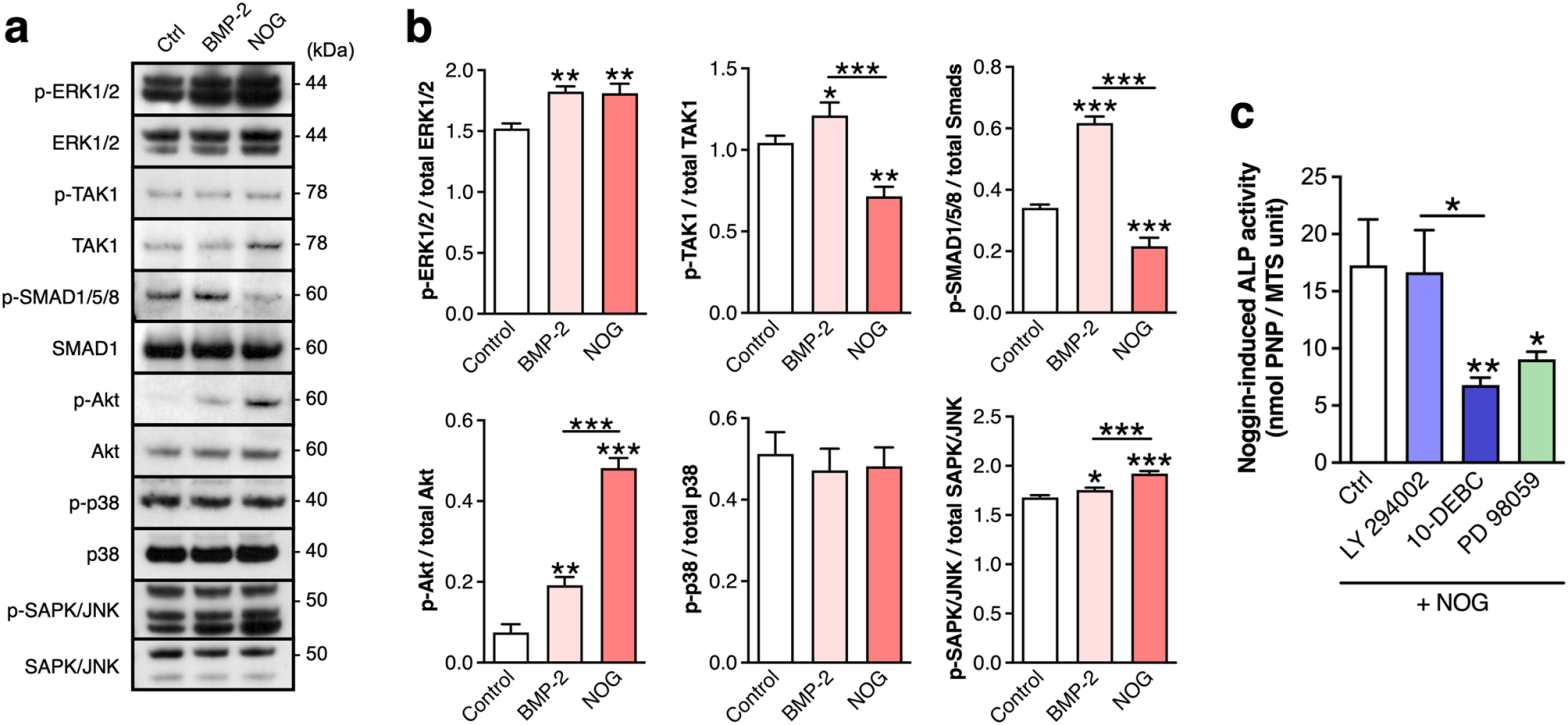
Noggin stimulates ALP activity in ASC cells via ERK1/2 and Akt kinases activation. (**a**) Western blot (WB) analysis of selected intracellular signal transducers after 4-d culture of ASC cells stimulated with either Noggin (NOG) or BMP-2 in osteogenic media. (**b**) Densitometric quantification of WB results expressed as a ratio of the activated proteins compared to total proteins. (**c**) Alkaline phosphatase activity (ALP) after 7-d osteogenic culture of ASCs treated with Noggin. On day 4 inhibitors of PI3-kinase (LY 294002), Akt (10-DEBC) or ERK1/2 (PD 98059) were added 1 h ahead of Noggin stimulation. (**a–c**) Control represents cells maintained in osteogenic medium containing ascorbic acid and dexamethasone. Average values ± SD are plotted. One-way ANOVA tests, *p < 0.05, **p < 0.001, ***p < 0.0001 relative to respective control or between marked groups.

Our results indicate that Noggin osteogenic capabilities extend to human adipose-derived stem cells (Fig. 2). Recently, it has been suggested that Noggin elevates ALP activity in human osteoblasts by binding competition to BMP receptors^36^. BMPs signaling activates either SMAD1/5/8 or the transforming growth factor activated kinase (TAK1), followed by p38, JNK and ERK1/2 activation^46,47^. We assessed the activation of the above-mentioned signal proteins upon Noggin or BMP-2 treatment. Western blot analyses revealed that ASC cells treated with Noggin increased phosphorylation of ERK1/2, and decreased phosphorylation of TAK1 and SMAD1/5/8 compared to BMP-2 treated ASC osteogenic cultures (Fig. 3a,b). BMP-2 treated cells activated both TAK1 and SMAD1/5/8. Thus, our results suggest that in ASC cells Noggin may signal through ERK1/2, independently of TAK1 and SMAD1/5/8. Furthermore, Noggin treatment of ASC cells resulted in increased levels of phosphorylated Akt and SAPK/JNK compared to either BMP-2 treated, or cells maintained in osteogenic medium only. To assess whether activation of Akt and ERK1/2 is necessary for Noggin to stimulate ALP activity, at culture day 4 ASC cells were pretreated with inhibitors of PI3-kinase (LY 294002), Akt (10-DEBC) or MEK1/2 (PD 98059; applied to block downstream ERK1/2 activation), followed by Noggin treatment, and they were assessed for ALP activity at culture day 7. In the presence of Akt (10-DEBC) or ERK1/2 (PD98059) inhibitors, ALP activities were significantly reduced upon Noggin treatment (Fig. 3c). Thus, our results suggest that Noggin may exert osteogenic effects through Akt and ERK kinases activation.

### Noggin treatment of adipose-derived stem cells is associated with the activation of fibroblast growth factor receptor type 2, Src, Akt and ERK1/2 kinases

**Figure 4:**
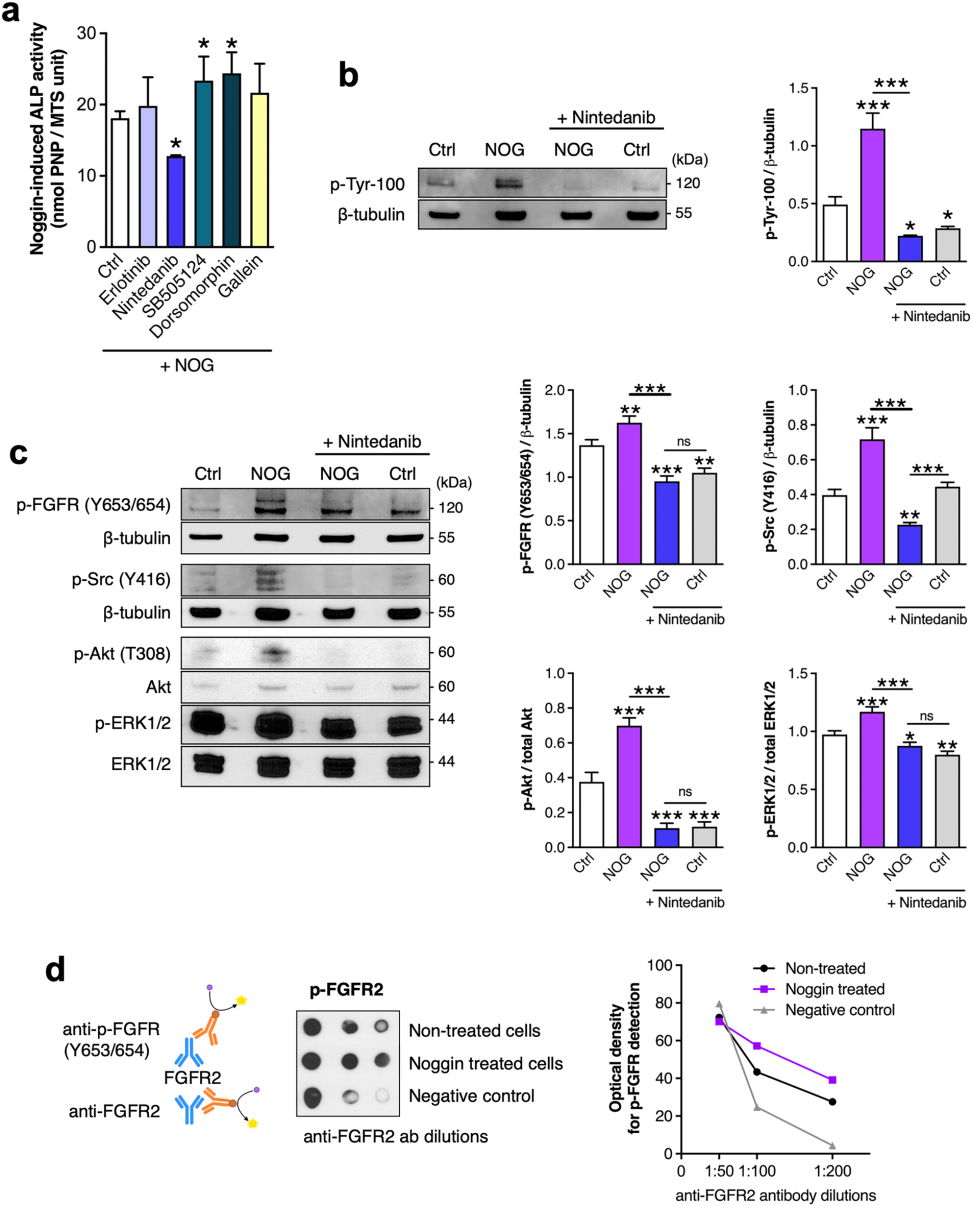
Noggin activates fibroblast growth factor receptor type 2 (FGFR2), Src, Akt and ERK kinases in ASC cells. (**a**) Alkaline phosphatase activity (ALP) after 7-d culture of ASC cells treated with Noggin (NOG) in osteogenic medium. On culture day 4 inhibitors of different type of receptors were added 1 h prior to Noggin stimulation. (**b**), (**c**) Western blot (WB) analysis of activated tyrosine kinases (p-Tyr-100), FGF receptors, Src, Akt and ERK1/2 kinases after 4-d culture of ASC cells. On culture day 4 cells were pretreated with Nintedanib 1 h prior to 1 h stimulation with Noggin. Control cells (Ctrl) were not treated with Noggin. WB results are presented in left panels. Densitometric quantifications of WB results expressed as a ratio of activated to total proteins are shown in right panels. (**d**) Phosphorylation of FGFR Type 2 (p-FGFR2) after 1-h Noggin stimulation of SaOS-2 cells, that are abundant in FGFRs^49^. Anti-FGFR2 antibodies were applied in dilutions on a membrane and probed with the equal amounts of whole cell extracts. This was followed by addition of antibodies detecting phosphorylated Y653/654 residues in FGFRs. For negative control the whole cell extracts were not added. Please note, that because both primary antibodies were raised in rabbits, secondary antibodies capture both primary antibodies. A control without extracts shows the level of diluted anti-FGFR2 antibodies on the membrane. Right panel shows densitometry results from the p-FGFR2 detection. (**a**–**d**) Quantitative data: average values ± SD are plotted. One-way ANOVA tests, *p < 0.05, **p < 0.001, ***p < 0.0001 relative to respective control or between marked groups.

To elucidate the putative adipose-derived stem cell surface receptor that is activated by Noggin and consequently activates intracellular Akt and ERK kinases (Fig. 3) we have first analyzed ALP activity of Noggin-treated ASC cells with and without chemical inhibitors for EGFR (Erlotininb), VEGFR, FGFR, PDGFR and Src kinase (Nintedanib), TGF-β receptors (SB505124), BMP receptors (Dorsomorphin) and G-protein coupled receptors (Gallein). These analyses identified Nintedanib as the only inhibitor that decreased Noggin-induced ALP activity at day 7 ASC cultures (Fig. 4a). Since Nintedanib can inhibit tyrosine kinase receptors, namely VEGFR, FGFR, PDGFR, we used antibodies against phosphorylated tyrosines (p-Tyr-100) to determine the molecular weight of the receptors activated by Noggin in ASC cells. As shown in Fig. 4b, Noggin stimulated phosphorylation of receptor tyrosine kinases of approx. 120 kDa molecular weight and Nintedanib markedly decreased this Noggin-induced phosphorylation. The molecular weight of 120 kDa suggested the activation of FGFRs by Noggin stimulation. Indeed, we observed increased FGFRs phosphorylation (Y653/654) along with increased phosphorylation of Src kinase (Y416), Akt and ERK1/2 after 1-h Noggin treatment of ASC cells (Fig. 4c). Furthermore, after treatment of cells with Nintedanib ahead of Noggin stimulation, Noggin effects on the activation of FGFR/Src/Akt and ERK1/2 signaling pathway were abolished (Fig. 4c). Since different types of FGFRs play opposite roles in osteogenesis^48^, we focused on Noggin effect on the FGFR type 2 phosphorylation using the osteosarcoma SaOS-2 cell model. These cells are commonly considered as osteoblastic cells and they are known for high FGFR2 expression levels^49^. These analyses showed that Noggin treatment of SaOS-2 cells resulted in increased phosphorylation of FGFR2 compared to non-treated cells (Fig. 4d). Taken together, we revealed for the first time that Noggin enhances phosphorylation of FGFR2 in both adipose-derived stem cells and osteosarcoma-derived osteoblastic cells and activates FGFR2/Src/Akt and ERK intracellular signaling pathway in adipose-derived stem cells.

**Figure 5:**
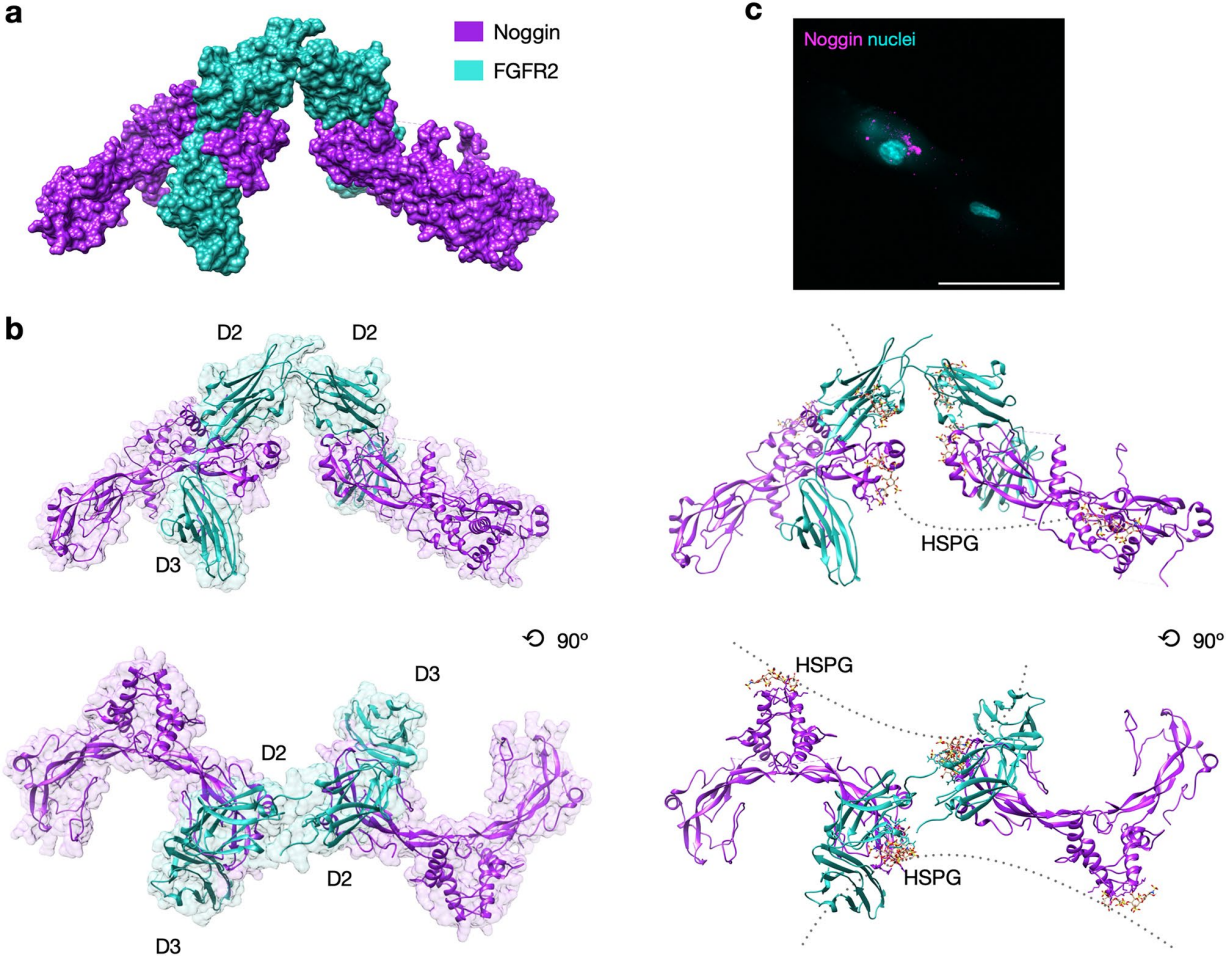
Hypothesized Noggin binding to the cell surface receptor FGFR2. (**a**) Molecular docking simulation of Noggin protein (PDB ID: 1M4U) and fibroblast growth factor receptor type 2 (PDB ID:1E0O). Results obtained with the use of ClusPro web server and visualized in Chimera. (**b**) Organization of FGFRs and Noggin proteins (cartoon representation) with heparin molecules (yellow stick representation) and possible heparan sulfate proteoglycans (HSPGs) connection within FGFR2-Noggin complexes (gray line). D2, D3—extracellular Ig domains of FGFR2. (**c**) Representative image of Noggin protein retained at the cell surface after ASC treatment with Noggin protein labelled with fluorescent dye CF 488A, Succinimidyl Ester (magenta colored). Cells nuclei stained with Hoechst (cyan colored). Confocal microscope, scale bar represents 100 μm.

To gain additional insights into potential Noggin binding to FGF receptors, we applied molecular docking simulation for docking Noggin protein with putative FGF receptor types 1–3. Docking simulation models in ClusPro web server were obtained by balanced scoring function including electrostatics, hydrophobicity and van der Waals interactions. We then assessed the activated models based on the assumption of Noggin localization within the FGF binding site and the model of Noggin-FGFR type 2 was selected (Fig. 5a). Since the binding of heparan sulfate proteoglycans (HSPGs) to Noggin protein is necessary for FGFR activation^50,51^, we have predicted HSPGs binding sites in Noggin protein. They were localized in the sites of the dimerization domain and near the finger domain, i.e., BMPs binding site (Fig. 5b). To verify whether Noggin can bind to ASC cell surface, we labeled Noggin protein with the fluorescent dye CF 488A, Succinimidyl Ester, which reacts with proteins amine groups. One hour after cells treatment with labelled Noggin, we observed accumulation of fluorescence signal on the cell surface (Fig. 5c, magenta colored) implying that Noggin can be retained at the ASC cell surface.

### Noggin enhances TAZ stabilization

**Figure 6:**
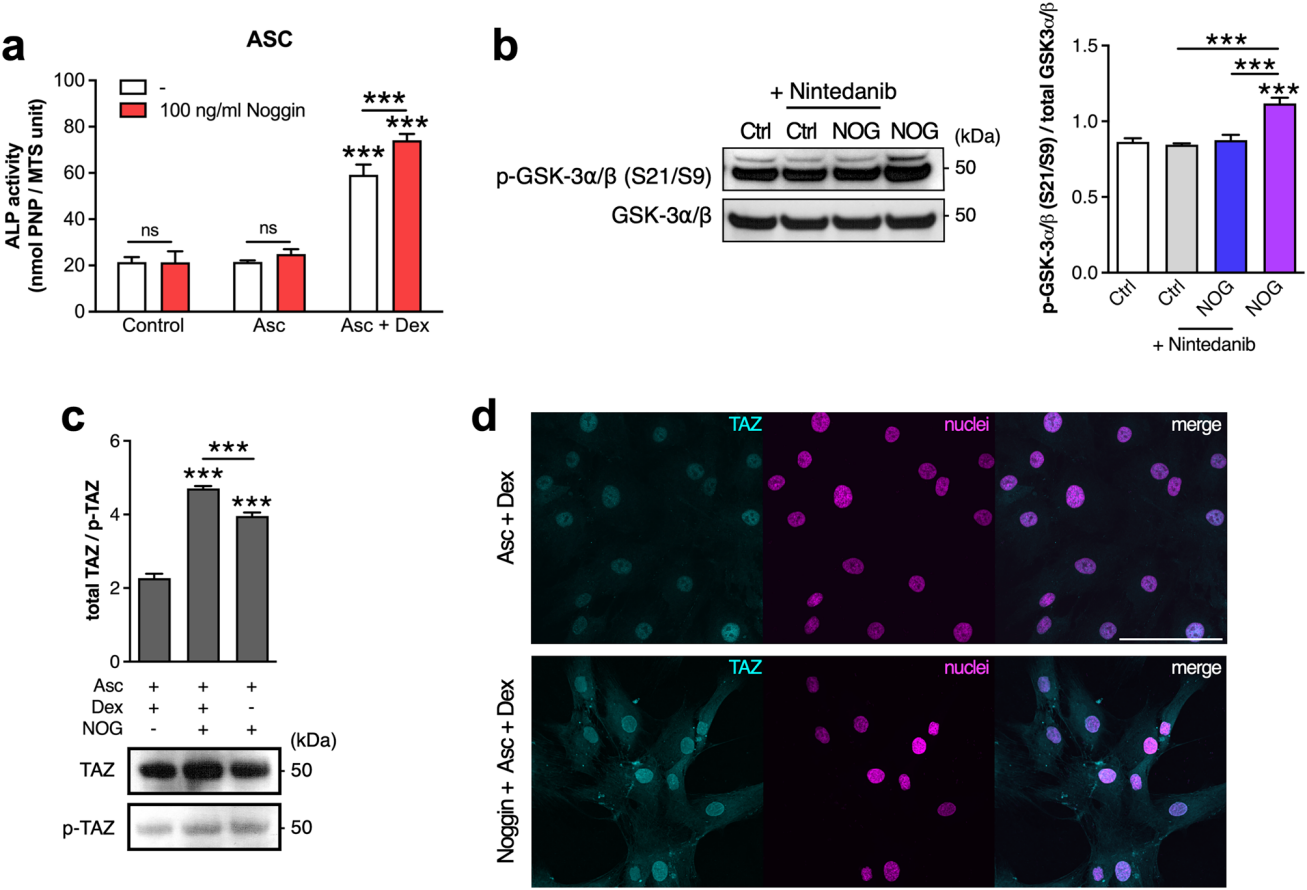
Noggin treatment results in TAZ accumulation in ASC cells. (**a**) Alkaline phosphatase activity of ASCs after 7-d treatment with Noggin (red bars) in standard culture media (control group) or supplemented with ascorbic acid (Asc) or ascorbic acid and dexamethasone (Asc + Dex). The white bars represent respective treatments without Noggin. (**b**) Western blot (WB) analysis of phosphorylated and total GSK-3⍺/β after 4-d culture of ASC cells. On culture day 4 cells were pretreated with Nintedanib 1 h prior to 1-h stimulation with Noggin. Control cells (Ctrl) were not treated with Noggin. (**c**) Western blot analysis of total TAZ and phosphorylated TAZ (p-TAZ) levels in 4-d culture of ASC cells treated with Noggin in either Asc + Dex supplemented medium or Asc supplemented only. On culture day 4 cells were treated with Noggin for 1 h and the whole cell extracts were analyzed. (**b–c**) Plots represent densitometric quantifications of WB results expressed as a ratio of examined proteins, as shown in blots. (**a–c**) Average ± SD are plotted. Two-way and one-way ANOVA tests, *p < 0.05, **p < 0.001, ***p < 0.0001, ns—not significant, relative to respective control or between marked groups. (**d**) TAZ localization in ASC cells (Alexa Fluor 488, cyan colored) after 3-day culture treatment with Noggin in osteogenic medium containing dexamethasone and ascorbic acid. Cell nuclei stained with DAPI (magenta colored). Confocal microscope, scale bar represents 100 μm.

Since Noggin treatment enhanced osteogenesis of ASC cells cultured in osteogenic media containing ascorbic acid and dexamethasone (Figs. 1 and 2), we also addressed the role of osteogenic media supplements in Noggin-stimulated ALP activity. We determined that Noggin increased cellular ALP activity only in the presence of dexamethasone and ascorbic acid, but not with ascorbic acid alone (Fig. 6a). Therefore, we focused on the potential link between Noggin and dexamethasone in inducing osteogenic events. We hypothesized that Akt kinase activated by Noggin (Fig. 4c) may inhibit GSK3 kinase^52^, which further blocks RUNX2 activity^53^ and sets TAZ proteins for degradation by their phosphorylation^54^. It is known that *TAZ* expression is up-regulated by dexamethasone treatment^55^ and TAZ protein is important for the formation of RUNX2-TAZ complexes that activate transcription of osteoblastic genes^56,57^. Thus, we analyzed the levels of phosphorylated and total GSK-3⍺/β and TAZ proteins in ASC cells treated with Noggin. Our results show that Noggin treatment leads to inactivation of GSK-3⍺/β kinases by their phosphorylation (S21/S9), compared to control cells or cells treated with Nintedanib (Fig. 6b). We also detected increased TAZ protein accumulation in cells treated with both Noggin and dexamethasone compared to dexamethasone alone (Fig. 6c). Finally, we observed increased TAZ protein levels in cells treated with Noggin, with the vast majority of Noggin protein accumulating in cell nuclei (Fig. 6d). Our results suggest that Noggin enhances stabilization of TAZ protein through blocking the activity of GSK-3⍺/β kinases, and thus preventing TAZ degradation. Taken together, Noggin appears to activate FGFR2/Src/Akt and ERK signaling pathways (Figs. 3, 4 and 5) and when added along with dexamethasone, it leads to TAZ protein stabilization (Fig. 6) and overall osteogenic differentiation of ASC cells (Fig. 2).

## Discussion

**Figure 7:**
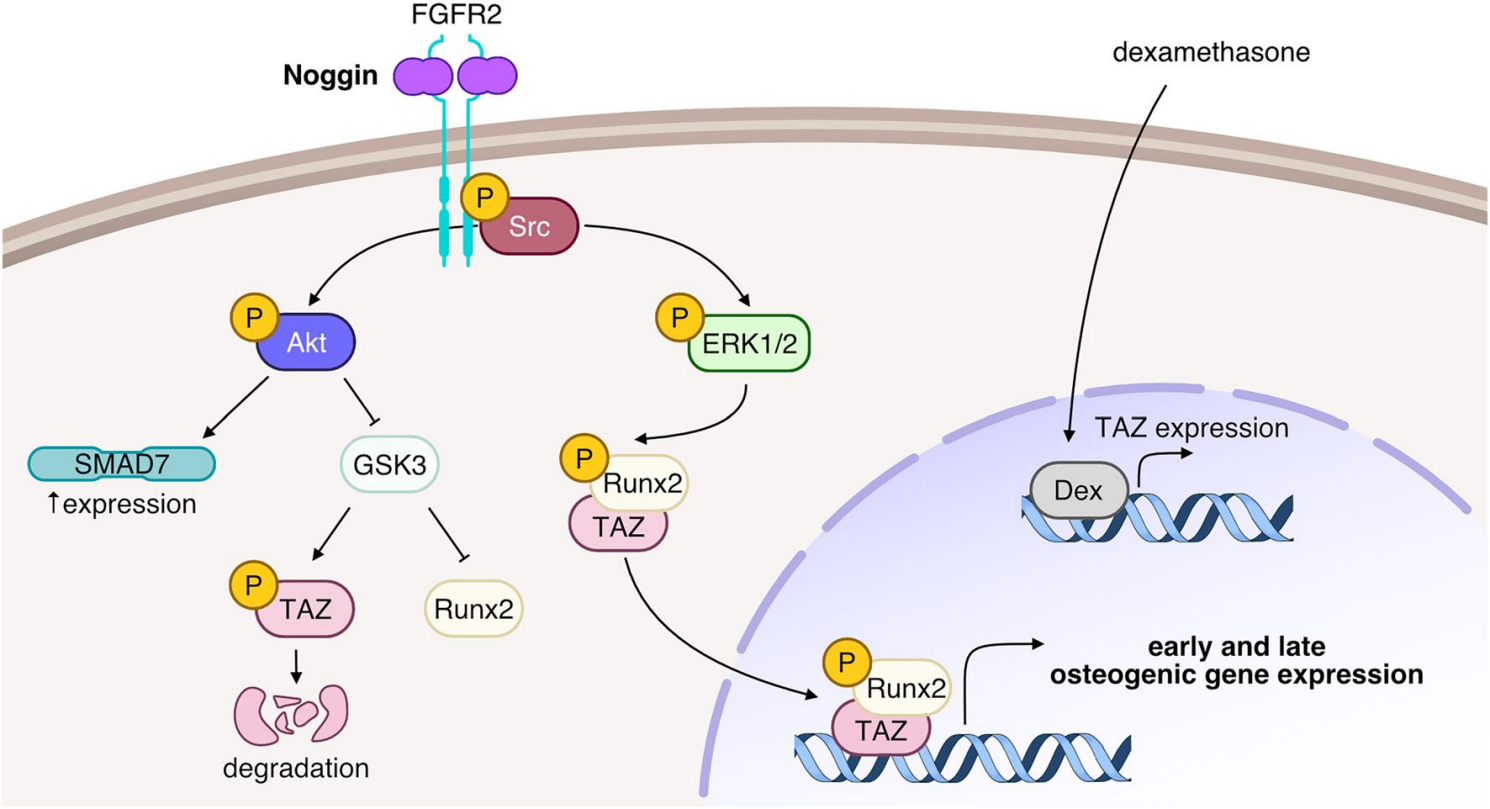
The suggested new signaling pathways induced by Noggin in human ASC osteogenic cultures. We have demonstrated that Noggin can activate FGFR2 receptors and Src kinase associated with the receptor complex. This results in ERK1/2 phosphorylation and, independently of PI3k, Akt kinase phosphorylation. It is known that dexamethasone, a component of osteogenic medium, stimulates *RUNX2* and *TAZ* expressions. We have shown Noggin activation of Akt that leads to blocking the ability of GSK3 to degrade TAZ and suppress RUNX2 activity, thereby stabilizing TAZ and enhancing formation of RUNX2-TAZ complexes. Whereas RUNX2-TAZ complexes can be phosphorylated by Noggin-activated ERK1/2. Such activated RUNX2-TAZ complexes are required for the transcription of osteogenic genes. Besides, we have shown Noggin-related inhibition of SMAD1/5/8 activity, which may be a result of increased *SMAD 7* expression due to Akt activity. Figure was created in Affinity Designer software (1.10.6).

In this study, we explored the possibility that Noggin may play a positive role in osteogenesis of human adipose-derived stem cells and potentially other human mesenchymal stem cells. We show, for the first time, a novel intracellular signaling pathway activated by Noggin (Fig. 7) for better understanding of Noggin molecular actions associated with bone-related cellular therapies.

We determined that Noggin increases ALP activity, an early marker of osteogenic cell progression, not only in osteoblasts^36^, but also in adult human mesenchymal stem cell populations derived from bone marrow, dental pulp and adipose tissue (Fig. 1a–c). Noggin also stimulates the osteogenic gene expression in cultures of normal human ASCs and BMSCs (Fig. 1e,f). Our results further indicate that continuous treatment of ASC cells with Noggin results in enhanced expression of both early and late osteogenic markers (Fig. 2a,b), and eventually robust extracellular matrix mineralization (Fig. 2c,d). To our knowledge, the effect of Noggin on mineral deposition in adult stem cells, was previously reported only in cultures of canine DPSC cells, but not in canine BMSCs^35^, though the exact mechanism of Noggin action was not explored. We determined that a dose of 100 ng/ml Noggin was sufficient to effectively induce ASC osteogenesis (Fig. 2). On the contrary, the same dose of BMP-2 had limited osteogenic effects in ASC cells (Fig. 2) consistent with previous reports regarding BMP applications in vitro^16–19^ as well as the requirements of higher doses of BMP-2 to achieve clinical efficacy^58^. We anticipate that our present findings open the possibility to use Noggin, instead of BMP, to effectively enhance osteogenic differentiation of adult human stem cells.

As the molecular mechanisms by which Noggin promotes osteogenesis remain unclear, we have explored the potential Noggin signaling pathways in ASC cells. Recently, Noggin and BMP-2 receptor binding competition was suggested in human osteoblast^36^ and Noggin was proposed to serve as a molecular switch controlling the fate of MSCs and inducing adipogenesis^37^. Given the above, we determined that Noggin treatment of ASC osteogenic cultures led to downregulation of BMP signaling, which was reflected by decreased SMAD1/5/8 and TAK1 phosphorylation (Fig. 3a,b). Reduced SMAD1/5/8 phosphorylation upon Noggin treatment was also reported in pancreatic beta cells, where Noggin inhibited apoptosis and promoted insulin secretion under high-glucose conditions^38^. Furthermore, treating human ASCs with Dorsomorphin, the inhibitor of BMP receptors, did not affect Noggin-induced ALP activity (Fig. 4a) and Noggin treatment did not result in BMP detection in the culture medium, opposite to BMP treated cells (Supplementary Fig. 1). Thus, Noggin effects do not appear to be due to downstream upregulation of BMP-2. Notably, our analyses also showed that Noggin stimulation of ASC osteogenic cultures resulted in increased phosphorylation of Akt (Thr308) and ERK1/2 kinases (Fig. 3a,b). Decreasing the activity of Akt and ERK1/2 kinases by selective chemical inhibitors (10-DEBC or PD 98059 respectively), significantly lowered Noggin-induced ALP activity, except for LY 294002 targeting the PI3k kinase that had no effect (Fig. 3c). Considering that activated Akt may inhibit Noggin expression by a feedback loop^59^, it is plausible that in ASC cells Noggin may negatively control its expression by activating Akt. Collectively, our data suggest that Noggin exerts its osteogenic effect in ASC cultures through phosphorylation of ERK1/2 and PI3k-independent activation of Akt.

Since we observed Akt, ERK1/2 and JNK activation followed by Noggin treatment of ASC cells (Fig. 3), we considered the potential involvement of receptor tyrosine kinases (RTKs), especially that recent studies imply that ancestor Noggin proteins were multifunctional cell regulators capable to activate RTK signaling pathways^60^. To explore this possibility, we applied inhibitors for EGFR (Erlotininb), VEGFR, FGFR, PDGFR (Nintedanib), G protein βγ subunit-dependent signaling (Gallein), BMP receptors (Dorsomorphin) and other types of TGF-β receptors (SB505124). We found that only Nintedanib decreased Noggin-induced ALP activity (Fig. 4a). Notably, Nintedaninb (BIBF 1120), used in this study at 1 nM concentration, inhibits only a narrow range of above-mentioned RTKs, all associated with Src kinase in their receptor complexes^61^. There are reports that some proteins, including Src kinase—a membrane associated non-receptor tyrosine kinase, restore Akt function without PI3k^62^ which is in agreement with our studies showing the PI3k-independent phosphorylation of Akt kinase (Fig. 3c). Targeting Src activity by Nintedanib abolished Noggin effect on Akt and ERK1/2 phosphorylation (Fig. 4c), which strongly suggests that Noggin-activated Src kinase may directly phosphorylate Akt and ERK1/2 kinases^63–65^.

Given that VEGFR, FGFR, PDGFR are tyrosine kinase receptors inhibited by Nintedanib, we determined that in ASC cells Noggin phosphorylates tyrosine residues of proteins of molecular weight corresponding to fibroblastic growth factor receptors (FGFRs; Fig. 4b). We further verified specific phosphorylation of tyrosine residues Y653/654 of FGFRs upon Noggin treatment (Fig. 4c). We also showed that Noggin activates FGF receptor type 2 in osteosarcoma cells SaOS-2 (Fig. 4d) that are known for high expression of FGFR subtypes^49^. Overall, we revealed that Noggin may activate FGFR2 followed by Src kinase activation that consequently leads to downstream Akt and ERK1/2 kinases phosphorylation. Besides, heparan sulfate proteoglycans (HSPGs) are necessary for FGF receptors activation. Given that they capture a wide range of growth factors, HSPGs facilitate ligand binding to the receptors^66^. HSPGs have also been shown to retain Noggin protein at the cell surface of CHO cells^3^, and we now show that Noggin can be retained at the ASC cells surface (Fig. 5c). It has been also suggested that Noggin may act in similar manner to Gremlin, another BMP antagonist. The latter binds to HSPGs, which facilitate its binding to VEGFR^4,66^. It is known that two FGF ligands bind FGFRs in the receptor binding-pocket oriented between D3 and D4 immunoglobulin-like extracellular motifs^67^ and HSPGs are required for this binding^68,69^. Our molecular docking simulation predicts that two Noggin protein molecules can bind to HSPGs and FGFR type 2 (Fig. 5b). It also reveals potential HSPG binding sites in Noggin molecule, i.e., at the dimerization domain and BMP binding finger domains. Although further studies, such as X-ray crystallography, are required to verify HSPGs-associated Noggin binding to FGFR. The molecular docking simulation together with our experimental data support the plausible interaction between Noggin and FGFR2 receptor.

This study supports the importance of FGFR2 and FGFR2-related intracellular signaling in osteogenesis of human adipose-derived stem cells. Previous reports demonstrated FGFR2 expression in osteoblasts and osteoprogenitor cells^70^, FGFR2/ERK pathway involved in osteogenesis of murine MSCs^71^, and *FGFR2* deletion causing the loss of mineral deposition by human MSCs^72^. Notably, there are also reports showing differentiation stage-specific inhibition of osteogenesis by FGF2 and Noggin in primary murine bone cell cultures^73^ and the requirement of BMP-2 for FGF-2 osteogenic action in newborn rat suture cells^74^. Thus, it is plausible there are species (i.e., rodent vs. human) differences in FGFR2 signaling. Especially that mutations in FGFR2 have also been linked to premature fusion of cranial sutures and skeletal disorders in humans, including Pfeiffer, Crouzon and Apert syndromes^51^. Thus, it seems of great relevance that Noggin may enhance osteogenesis of human ASCs through FGFR2-related signaling. Our studies also indicate that Noggin action in adult stem cells depends on the context of other, coexisting with Noggin, cell differentiation factors. Specifically, in the presence of osteogenic medium, Noggin may enhance ASC osteogenesis (Fig. 6), whereas in the presence of adipogenic medium it enhances MSC adipogenesis^37^. In view of the above, Noggin-favored adipogenic differentiation of human ASCs is also possible due to Noggin-activated FGFR2, as FGFR2 are expressed in adipocytes and FGF2 elevated signaling are found in obesity^75^.

To explore further the impact of osteogenic medium components on Noggin osteogenic action in ASC cells, we determined that Noggin increased ALP activity only in the presence of dexamethasone (Fig. 6a). Since dexamethasone can stimulate the expression of both *TAZ* and *RUNX2*^55,76^, we determined that TAZ protein levels were markedly increased upon Noggin and dexamethasone treatment (Fig. 6c), probably due to phosphorylation and thus inhibition of GSK-3⍺/β. In view of the potential interplay between TAZ and telomerase in telomerase-immortalized cell lines^77^, including ASC52telo cell line, we verified that Noggin increased early osteogenic markers in both human primary ASCs and BMSCs (Fig. 1e–f). In normal human ASCs we also detected the vast accumulation of TAZ protein in cell nuclei within consolidated nuclear envelope (Fig. 6d). Our results are consistent with recent reports showing that TAZ can preserve the nuclear envelope activity^78^ and generally TAZ proteins are associated with actin cytoskeleton as they are engaged in mechanotransduction^79^. We assume that Akt phosphorylation upon Noggin treatment may lead to stabilization of TAZ proteins that eventually enables the formation of RUNX2-TAZ transcriptional complexes^56,57^. Consequently, phosphorylation of ERK1/2 upon Noggin treatment (Fig. 3) can lead to activation of such RUNX2-TAZ complexes and result in osteoblastic genes expression (Fig. 2). It is well recognized that Akt phosphorylation and activation can lead to phosphorylation and thus inhibition of GSK3 activity^52^. Our results indicate that Noggin-treated ASCs activate Akt kinase and inactivate GSK-3⍺/β (Fig. 6b). The latter stimulates TAZ ubiquitylation and degradation^54^ and suppresses RUNX2 activity^53^. Thus, our data support the hypothesis that, in human ASC cultures, Noggin stabilizes TAZ proteins due to Akt activation, followed by GSK3 inhibition (Fig. 7). Besides, Akt kinase can also modulate TGF-beta/BMP signaling by increasing the expression of inhibitory SMAD7^80^, but this requires further studies. In our ASC osteogenic cultures, Noggin decreased levels of phosphorylated SMAD1/5/8 (Fig. 3a,b) similar to recent work that showed Noggin suppression of TGF-beta signaling by SMAD2/3 dephosphorylation^81^. It is plausible that SMAD7 inhibition of SMAD1/5/8 could have been involved in Noggin osteogenic action in human ASC cells. Furthermore, the activation of Akt (T308) may lead to decreased mTORC1 activity, and result e.g., in the angiogenic VEGF expression^82^. We also observed increased *VEGF* mRNA levels under Noggin treatment, that suggests the potential involvement of mTORC1 deactivation, but this needs further verifications.

Collectively, our present findings demonstrate that in osteogenic cultures of human adipose-derived stem cell cultures Noggin enhances both early and late osteogenic markers expression and promotes extracellular matrix mineralization, but only if the Noggin treatment is accompanied by dexamethasone, a component of standard osteogenic medium. The underlying mechanisms of Noggin osteogenic action involve the activation of FGF receptor type 2 and membrane bound Src kinase, followed by the activation of intracellular Akt and ERK1/2 kinases. The up-regulation of *TAZ* expression by dexamethasone, together with the stabilization of the TAZ-RUNX2 complexes due to the action of activated Akt and ERK1/2, promote osteogenic progression of human ASCs (Fig. 6). We thus provide an alternative strategy to stimulate osteogenesis of human adipose-derived stem cells using Noggin and dexamethasone. Considering controversies regarding off-label BMP-2 clinical applications that result in several unexpected side effects^9–15^, ASC treatment with Noggin and dexamethasone may prove useful in various bone-related cellular therapies that involve adipose-derived stem cells. We are aware that the present in vitro findings need in vivo verifications. Yet, most studies dealing with Noggin have focused on its inhibition to increase BMP osteogenic action^30,31,33^, whereas some use Noggin treatment to inhibit unwanted BMP-induced ossification^26,27^. The appropriate Noggin levels in vivo may also matter, as Noggin inactivation reportedly caused osteopenia in mice^34^. Overall, while these studies focused on the modulation of BMP signaling, we now propose that a surplus of Noggin over the BMP can be added along with dexamethasone, to assess Noggin potential implications in vivo. The Noggin-induced FGFR2/Src/Akt/ERK signaling pathway, revealed in our study, opens a new route for further exploration of the Noggin action other than modulation of BMP signaling and its potential application in therapies that require bone regeneration. We believe our findings advance our knowledge on Noggin intracellular signaling and its pleiotropic role in modulating the adult stem cells fate.

## Methods

### Cells and culture media

ASC52telo cells (ASC; ATCC, SCRC-4000) and normal human ASC (ATCC, PCS-500-011) were expanded in the dedicated medium (ATCC, Mesenchymal Stem Cell Basal Medium with Mesenchymal Stem Cell Growth Kit). For experiments, cells were switched to complete growth medium consisting of 89% MEM Alpha (ThermoFisher Scientific), 10% FBS Q (Biological Industries) and 1% ZellShield antibiotics (Minerva Biolabs). Human bone marrow stem cells (BMSC) were obtained from the iliac crest and dental pulp stem cells (DPSC) from the molar tooth of adult donors according to the approved institutional review board protocol no. 1072.6120.253.2017. BMSC and DPSC were expanded in complete growth medium and used for experiments at passages 4–7. Cell cultures were maintained in culture incubator at 37 °C, 5% CO_2_ humidified atmosphere; culture media were exchanged every 2–3 days and cells were passaged using 0.25% trypsin/EDTA (ThermoFisher Scientific) before they reached full confluence. For experiments, ASC cells were seeded at the density of 1000/cm^2^, BMSC and DPSC at the density of 10,000/cm^2^.

### Experimental cell culture treatments

Osteogenic medium containing complete growth medium supplemented with 100 μg/ml ascorbic acid, 10^−7^ M dexamethasone and 10 mM β-glycerophosphate (all from Sigma-Aldrich) was applied from day 1 cultures. Recombinant human Noggin (rhNoggin, Origene) or recombinant human BMP-2 (rhBMP-2, ThermoFisher Scientific) were added from day 1 cultures at concentrations of 100 ng/ml, unless indicated otherwise. The media and supplements were exchanged every 3 days. Chemical inhibitors of the receptors and intracellular kinases were purchased from R&D systems or from Sigma Aldrich and used at the following final concentrations: 2 μM Erlotinib (EGFR), 1 nM Nintedanib (VEGFR, PDGFR, FGFR, and Src), 10 μM SB505124 (TGFβRI), 5 μM Dorsomorphin (BMPRI), 10 μM Gallein (GPCR), 10 μM LY 294002 (PI3-kinase), 2,5 μM 10-DEBC (Akt) and 50 μM PD 98059 (MEK1/2 inhibitor; applied to block downstream ERK1/2 activation). The chemical inhibitors were added on culture day 4, 1-h prior to Noggin additions.

### ALP activity

ALP activity was examined using a substrate p-nitrophenol phosphate (PNP, Sigma-Aldrich). Cell cultures were first assayed for viability using The CellTiter 96 AQueous One Solution Cell Proliferation Assay (Promega) and then protein extracts were obtained with the Cell Digestion Buffer consisting of 89% dH_2_O, 10% Cell Assay Buffer (CAB) and 1% Triton X-100. The CAB consisted of 91.2 g Tris, 0.102 g MgCl_2_ × 6 H_2_O and 0.068 ZnCl_2_ per 500 ml dH_2_O^16^. The protein extracts were combined at 1:9 ratio with the PNP dissolved at 37.1 mg per 20 ml CAB. Absorbance changes per minute were measured spectrophotometrically at 405 nm using SpectraMax iD3 Molecular Devices reader. The results were normalized to viable cell number.

### Mineralization of extracellular matrix

Cell cultures were fixed with 100% methanol and then stained for 30 min with 1% (water solution) Alizarin Red S (ARS). For semi-quantitative assessment of ECM mineralization level, the plates were washed with distilled water and the ARS extracted with 5% perchloric acid. The absorbance of the extracted dye was measured at 490 nm using SpectraMax iD3 Molecular Devices reader. The results were normalized to viable cell number, as described above.

### RT-PCR

Total RNA was extracted using TRI Reagent (Zymo Research). Equal amounts of RNA were reverse transcribed using high-capacity cDNA Reverse Transcription kit (Applied Biosystems). The PCR amplifications were performed using the StepOnePlus Real-Time PCR Systems (Applied Biosystems). Each reaction mixture contained 50 ng of cDNA, 2–3 µM forward and reverse primers (Metabion) with Sensitive RT HS-PCR Mix SYBR (A&A Biotechnology) or TaqMan probes with TaqMan Universal Master Mix (Thermofisher Scientific). The following probes were used: RUNX2 Hs00231692_m1, SP7 (Osterix) Hs01866874_s1, TBP Hs00427620_m1. The applied primer sets were as follows: Osteocalcin (OC), forward: 5’-AAGAGACCCAGGCGCTACCT-3’ and reverse: 5’-AACTCGTCACAGTCCGGATTG-3’; Bone sialoprotein (BSP), forward: 5’-AACGAAAGCGAAGCAGAA-3’ and reverse: 5’-TCTGCCTCTGTGCTGTTGGT-3’; Osteopontin (OPN), forward: 5’-TGGAAAGCGAGGAGTTGAATG-3’ and reverse: 5’-CATCCAGCTGACTCGTTTCATAA-3’; Osteoprotegerin (OPG), forward: 5’-GTCAAGCAGGAGTGCAATCG-3’ and reverse: 5’-TAGCGCCCTTCCTTGCATT-3’; Osteonectin (ON), forward: 5’-GACTACATCGGGCCTTGCAA-3’ and reverse: 5’-GGGAATTCGGTCAGCTCAGA-3’; Vascular endothelial growth factor (VEGF), forward: 5’-GAGTGTGTGCCCACTGAGGAGTCCAAC-3’ and reverse: 5’-CTCCTGCCCGGCTCACCGCCTCGGCTT-3’; Collagen type I (COL1A1), forward: 5’-GTCTAGACATGTTCAGCTTTGTGGA-3’ and reverse 5’-CTTGGTCTCGTCACAGATCACGTCAT-3’; Bone morphogenetic protein 2 (BMP-2), forward: 5’-TGCTAGTAACTTTTGGCCATGATG-3’ and reverse: 5’-GCGTTTCCGCTGTTTGTGTT-3’; Noggin (NOG), forward: 5’GCGCTGCGGCTGGAT-3’ and reverse: 5’-AGCACTTGCACTCGGAAATGA-3’; TBP, forward: 5’-GGAGCTGTGATGTGAAGTTTCCTA-3’ and reverse: 5’-CCAGGAAATAACTCTGGCTCATAAC-3’. Relative expression levels were obtained with the 2^−ΔΔCT^ method.

### Western blot

The whole-cell extracts were obtained using cell lysis buffer (Cell Signaling Technology). Protein concentrations were determined by Pierce MicroBCA Protein Assay Kit (ThermoFisher Scientific). Equal amounts (50 μg) of proteins were separated on NuPAGE 4–12% Bis–Tris gels and transferred to PVDF membranes (ThermoFisher Scientific). Membranes were probed overnight with primary anti-human antibodies and then for 1 h with the horseradish peroxidase-conjugated secondary goat anti-rabbit or anti-mouse antibodies (Abcam, ab6721, ab6728). The following primary antibodies (Cell Signaling Technology, CST) were used (all dilutions as recommended by CST): mouse anti-p-tyrosines p-Tyr-100 (#9411) and rabbit: anti-p-ERK (#9101), anti-ERK (#9102), anti-p-TAK1 (#9339), anti-TAK (#4505), anti-p-SMAD1/5/8 (#13820), anti-SMAD1 (#6944), anti-p-Akt (#4056), anti-Akt (#9272), anti-p-38 (#4511), anti-p38 (#8690), anti-p-SAPK/JNK (#4668), anti-SAPK/JNK (#9252), anti-p-Src (#6943), anti-p-FGFR (#3471), anti-FGFR2 (#23328), anti-p-TAZ (#59971), anti-TAZ (#70148). Rabbit anti-GSK-3 ⍺/β and anti-p-GSK-3 ⍺/β antibodies were obtained from R&D Systems DuoSet (DYC2630-2). Rabbit anti-β-tubulin (Abcam, ab6046) antibodies were used for normalization. For p-FGFR detection rabbit anti-FGFR2 antibodies were applied in indicated dilutions to a PVDF membrane and probed with the equal amounts (100 μg) of whole cell extracts. This was followed by addition of antibodies detecting phosphorylated Y653/654 residues in FGFRs (rabbit anti-p-FGFR). Secondary goat anti-rabbit antibodies (Abcam, ab6721) were used for detection. The signal was developed by Western Lightning Chemiluminescence Reagent Plus (GE Healthcare) and the results were generated on CL-XPosure Film (ThermoFisher Scientific).

### Molecular docking simulation

Structure based protein–protein docking was performed in ClusPro web server^83–85^. Simulation of potential molecular interactions was made for Noggin protein (PDB ID:1M4U) and extracellular Ig domains of fibroblast growth factor receptors type 1 (PDB ID:1FQ9), 2 (PDB ID:1E0O) and 3 (PDB ID: 1RY7). Additionally, the location of heparin-binding sites on Noggin protein was assessed. Results of molecular docking simulation were visualized in Chimera.

### Fluorescent imaging

CF 488A, succinimidyl ester (CF488A SE, (Sigma-Aldrich) was used to label Noggin protein at dye:protein molar ratio of 15:1 according to manufacturer’s instructions. ASC cultures were treated with labeled Noggin for 1 h, followed by cell nuclei staining with Hoechst (Sigma-Aldrich). For TAZ localization, normal human ASC cells were treated for 3 days with dexamethasone, ascorbic acid and Noggin. At culture day 3, cells were treated for 1 h with Noggin, then fixed with 4% paraformaldehyde, permeabilized with 0.2% Triton X-100 and blocked with 5% serum. Cells were incubated with anti-TAZ (#70148) antibodies overnight, followed by probing with goat anti-rabbit secondary antibody conjugated with Alexa Fluor 488 (Invitrogen) for 1 h. Cell nuclei were stained with DAPI (Sigma-Aldrich). Images were acquired with a ZEISS LSM 880 Confocal Laser Scanning Microscope.

### Statistical analysis

All experiments were performed in triplicates, data were collected as mean +/− SD, and data were analyzed for statistical significance using one-way or two-way analysis of variance (ANOVA) followed by Tukey’s tests for multiple comparisons.

### Supplementary Information

Supplementary Figure 1.

Supplementary Information.

## Data Availability

The data that support the findings of this study are available from the corresponding author upon reasonable request.
