# Supplementary material for: Noggin promotes osteogenesis in human adipose-derived mesenchymal stem cells via FGFR2/Src/Akt and ERK signaling pathway
Source: Sci Rep. 2024 Mar 20;14:6724. doi: 10.1038/s41598-024-56858-w (PMC10954655; doi:10.1038/s41598-024-56858-w)
Supplement: Supplementary file 1 — Supplementary Figure 1. [file 41598_2024_56858_MOESM1_ESM.pdf]

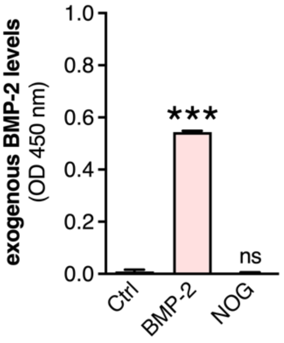

Supplementary Figure 1. The BMP-2 protein levels in the culture media at day 7 of ASC culture (ELISA kit for human BMP-2, Invitrogen). Cells were treated with either 100 ng/ml recombinant human Noggin (NOG) or 100 ng/ml recombinant human bone morphogenetic protein 2 (BMP-2), or left untreated (control, Ctrl) in osteogenic medium containing ascorbic acid (Asc) and dexamethasone (Dex). Average values ±SD are plotted. One-way ANOVA tests, ***p<0.0001, ns - not significant.
