# Supplementary material for: Noggin promotes osteogenesis in human adipose-derived mesenchymal stem cells via FGFR2/Src/Akt and ERK signaling pathway
Source: Sci Rep. 2024 Mar 20;14:6724. doi: 10.1038/s41598-024-56858-w (PMC10954655; doi:10.1038/s41598-024-56858-w)

Supplementary Information

Representative immunoblots shown in Figures 3-6. Some membranes were cut prior hybridization with antibodies and processed in parallel.

Figure 3a

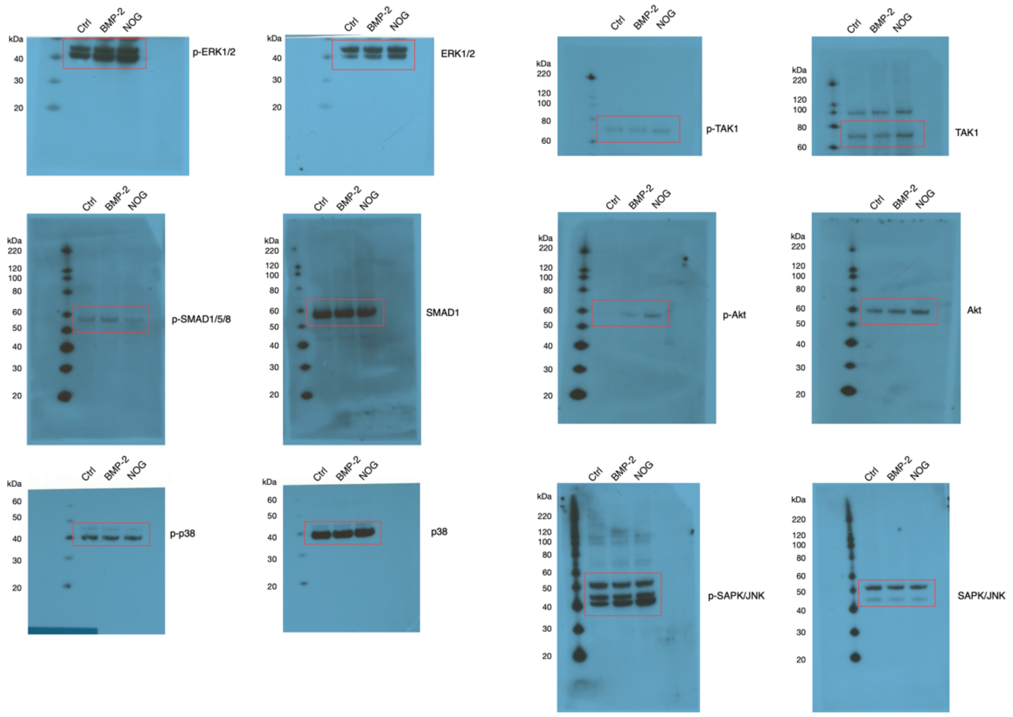

Figure 4b-d

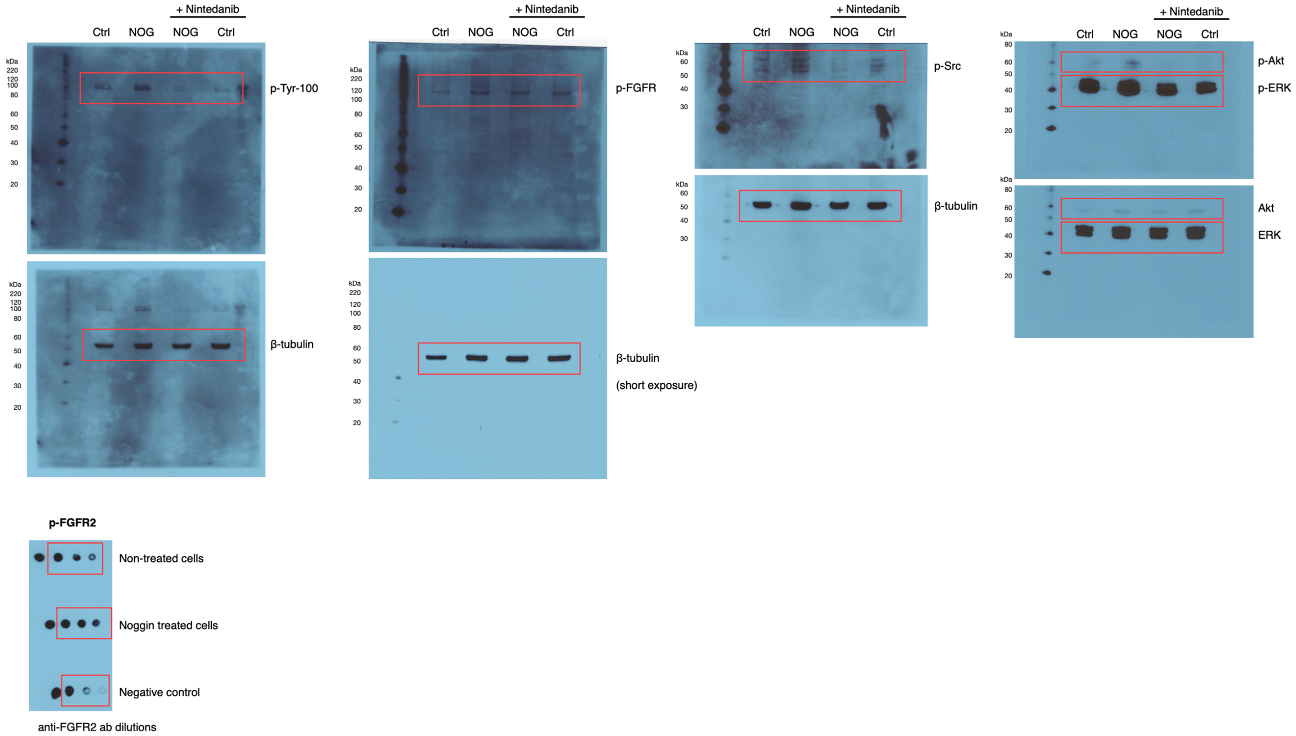

Figure 6 b-c

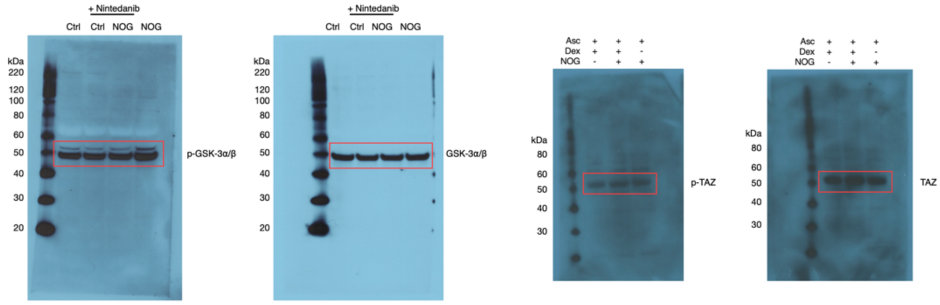

Supplement: Supplementary file 2 — Supplementary Information. [file 41598_2024_56858_MOESM2_ESM.pdf]
